# Supplementary material for: A Critical View of the Application of the APEX Software (Aqueous Photochemistry of Environmentally-Occurring Xenobiotics) to Predict Photoreaction Kinetics in Surface Freshwaters
Source: Molecules. 2019 Dec 18;25(1):9. doi: 10.3390/molecules25010009 (PMC7017383; doi:10.3390/molecules25010009)
Supplement: Supplementary file 1 [file molecules-25-00009-s001.zip › 3.2.4_gcc-4.4.0.zip!/3.2.4_gcc-4.4.0/3.2.4_gcc-4.4.0/doc/octave/html/interpreter/Creating-Strings.html]

Creating Strings - Untitled


Next: Comparing Strings,
Previous: Character Arrays,
Up: Strings


---

### 5.3 Creating Strings

The easiest way to create a string is, as illustrated in the introduction,
to enclose a text in double-quotes or single-quotes. It is however
possible to create a string without actually writing a text. The
function `blanks` creates a string of a given length consisting
only of blank characters (ASCII code 32).

— Function File: **blanks** (n)  
> Return a string of n blanks, for example:
>
> ```
>           blanks(10);
>           whos ans;
>                =>
>                 Attr Name        Size                     Bytes  Class
>                 ==== ====        ====                     =====  =====
>                      ans         1x10                        10  char
> ```
>
>
>
>
> **See also:** repmat.

- Concatenating Strings- Conversion of Numerical Data to Strings
